# Higher N-terminal pro-brain natriuretic peptide level at onset of peritoneal dialysis-related peritonitis is a risk factor for technique failure

**DOI:** 10.1186/s12882-024-03603-0

**Published:** 2024-05-17

**Authors:** Niya Ma, Zhiyun Zang, Xia Liu, Yunyun Zhang, Xueli Zhou, Yi Tang, Zi Li

**Affiliations:** 1 Department of Nephrology, West China Hospital of Sichuan University, Guoxue Lane 37, Chengdu, 610041 China; 2 Department of Nephrology, The Third People’s Hospital of Chengdu, Chengdu, 610014 China; 3 West China School of Nursing, West China Hospital of Sichuan University, Chengdu, 610041 China

**Keywords:** N-terminal fragment of B-type natriuretic peptide, Outcome, Peritoneal dialysis-associated peritonitis

## Abstract

**Background:**

Recent studies have suggested that the N-terminal fragment of B-type natriuretic peptide (NT-proBNP) level serve as a significant risk factor for mortality in patients with end-stage renal disease. However, the relationship between NT-proBNP levels and technique failure in peritoneal dialysis-associated peritonitis (PDAP) remains unclear. This study investigated the relationship between NT-proBNP levels at the onset of PDAP and the risk of technique failure in patients with PDAP.

**Methods:**

A retrospective analysis was conducted on patients with PDAP from December 1, 2009, to December 31, 2021, at our peritoneal dialysis center. We recorded all demographic and baseline clinical data at the time of admission for each PDAP episode. Logistic and Cox regression analyses were performed to assess the association between NT-proBNP levels and technique failure.

**Results:**

Of 485 PDAP episodes included in this study, 130 episodes of technique failure were observed. Multivariate logistic analysis revealed that hospital stay, Na and NT-proBNP levels, and peritoneal dialysate white blood cell counts on days 3 and 5 were independently associated with technique failure. The receiver operating characteristic curve demonstrated that the NT-proBNP level was a better indicator than the other four variables in indicating technique failure. In the multivariate Cox regression analysis, after adjusting for confounding factors, higher NT-proBNP levels (HR of 3.020, 95% CI 1.771, 5.150, *P* < 0.001) were associated with PDAP technique failure.

**Conclusions:**

This retrospective study identified the serum NT-proBNP level at the onset of PDAP as an independent risk factor for technique failure in these patients.

## Background

Peritoneal dialysis (PD) is a vital treatment for patients with end-stage renal disease (ESRD). However, peritonitis is a major complication of PD and the leading cause of technique failure. Peritoneal dialysis-associated peritonitis (PDAP) is a direct or major cause of death in 15% of patients undergoing PD [1, 2]. According to the Standardized Outcomes in Nephrology (SONG-PD) Initiative, PD-related infection has been identified as a core outcome of critical importance for patients and clinicians [3]. Therefore, it is important to accurately assess and identify the risk factors for peritonitis to prevent serious outcomes, including death.

Previous studies have focused on laboratory parameters to assess the risk of technique failure during PDAP, mainly focusing on coagulation and fibrin factors and serum albumin and C-reactive protein (CRP) levels [4–6]. The N-terminal fragment of B-type natriuretic peptide (NT-proBNP) is a biomarker of fluid volume overload and myocardial damage and has been used as a risk factor of mortality in patients with ESRD [7, 8]. Some studies have suggested that NT-proBNP may be a biomarker for estimating the risk of PD technique failure [9]. However, the association between NT-proBNP levels and technique failure in patients with PDAP is not yet understood.

The purpose of this study was to investigate whether the NT-proBNP level at admission for PDAP is associated with the incidence of technique failure in patients with PDAP. The findings of this study may provide new insights into the clinical management of patients with PDAP.

## Methods

### Study design and population

This single-center, retrospective study enrolled patients undergoing maintenance PD at the PD center of the West China Hospital, Sichuan University. Patients aged ≥ 18 years who were admitted due to PDAP between December 1, 2009, and December 31, 2021, were included in this study. PDAP was diagnosed in accordance with the criteria of the 2022 International Society for Peritoneal Dialysis (ISPD) guidelines. The exclusion criteria were as follows: (1) PD < 3 months, (2) missing data (absence of plasma NT-proBNP level, white cell count of peritoneal dialysate, or the results of pathogenic culture), and (3) loss to follow-up.

This study was approved by the Medical Ethics Committee of the West China Hospital of Sichuan University, Sichuan, China (2019-33) and was registered in the Thai Clinical Trials Registry (TCTR20180313004). This study adhered to the tenets of the Declaration of Helsinki, and written informed consent was obtained from all participants.

### Data collection

Clinical and laboratory data were collected from electronic medical records and laboratory information systems. Clinical characteristics such as age, sex, place of residence, PD vintage, systolic blood pressure (SBP), and diastolic blood pressure (DBP) were recorded. Comorbidities, including diabetes mellitus (DM) and cardiovascular diseases (CVDs), were recorded. The Charlson Comorbidity Index (CCI) score was calculated based on the patient’s age and comorbidities. Baseline laboratory data, collected at the first examination after *admission* due to PDAP, included hemoglobin (HB), white blood cell count (WBC), neutrophil count, platelet count (PLT), direct bilirubin (DB), indirect bilirubin (IB), serum albumin (ALB), serum globulin (GLB), blood glucose (GLU), blood urea nitrogen (BUN), serum creatinine (Scr), uric acid (UA), triglycerides (TG), total cholesterol (CHOL), low-density lipoprotein cholesterol (LDL-C), serum sodium (Na), serum potassium (K), serum calcium (Ca), serum phosphorus (P), intact parathyroid hormone (iPTH), activated partial thromboplastin time (APTT), prothrombin time (PT), fibrinogen (FIB), NT-proBNP, and ferritin levels. The peritoneal dialysate white cell counts on days 1, 3, and 5, and the causative organisms according to culture results were also collected. The dialysate sample for pathogen culture was collected just after admission to the hospital, prior to the administration of empirical antibiotic therapy in our hospital.

### Diagnostic criteria for PDAP and study outcomes

According to the Recommendations for the Prevention and Treatment of Peritonitis in ISPD: 2022 Update, PDAP was diagnosed when two or more of the following three factors were present: (1) abdominal pain and/or cloudy dialysis effluent; (2) dialysis effluent white cell count > 100/µL after a dwell time of at least 2 h, with > 50% polymorphonuclear leukocytes (PMN); and (3) positive dialysis effluent culture. According to the 2022 ISPD guidelines, patients with PDAP were allocated to one of two groups according to their clinical outcomes: (1) technique survival group: after 2–3 weeks of reasonable antibiotic treatment, the symptoms of peritonitis were completely relieved, the dialysate became clear, and the dialysate white blood cell count decreased to normal levels; (2) technique failure group: (a) peritonitis-related catheter removal; (b) temporary or permanent conversion to hemodialysis due to severe complications; and (c) death within 30 days of peritonitis onset or during hospitalization for peritonitis.

### Statistical analysis

Continuous variables are expressed as mean ± standard deviation or median (interquartile range). The unpaired t-test was used for normally distributed data and the Mann–Whitney U test for non-normally distributed data. Categorical variables, expressed as frequencies (percentages), were compared using the chi-square test. Significant variables identified by univariate analysis were further analyzed using binary logistic regression to identify the independent prognostic power of the indices. Receiver operating characteristic (ROC) curves were used to analyze and summarize factors affecting the prognosis of patients with PDAP. Multivariate Cox regression models were used to analyze variables that were statistically significant in the univariate analysis as well as variables considered to be associated with technical failure. All statistical analyses were performed using SPSS 26.0 (IBM Corp., Armonk, NY, USA). A two-sided *P* value less than 0.05 was considered statistically significant.

## Results

**Fig. 1:**
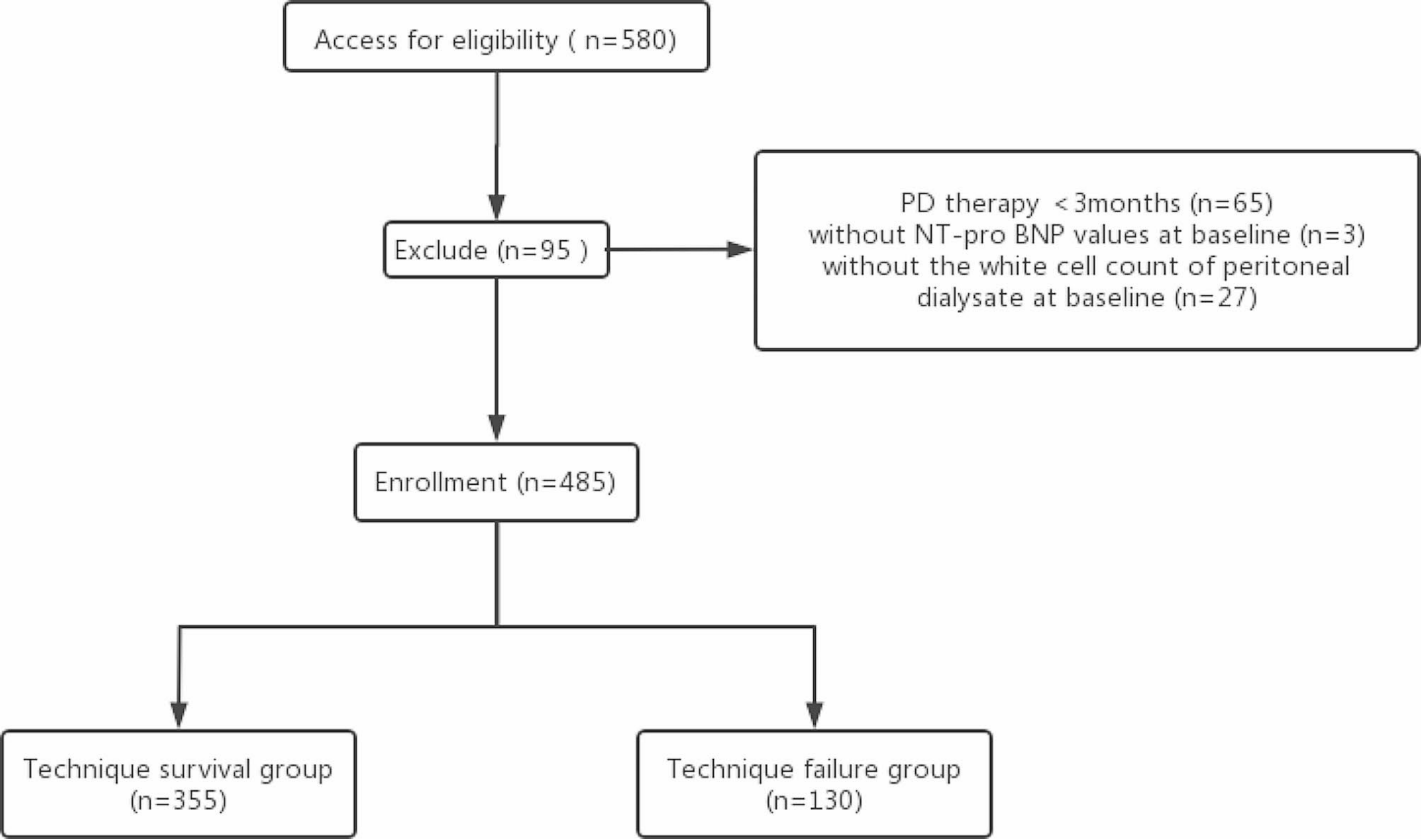
Flow chart of the participants in the study. PD, peritoneal dialysis

A total of 580 PDAP episodes were screened (Fig. 1). However, 95 episodes were excluded based on the following exclusion criteria: 3 episodes with missing data for the baseline NT-proBNP level, 27 episodes with missing data for the white cell count of the peritoneal dialysate, and 65 episodes with PD less than 3 months. The remaining 485 PDAP episodes were included in further analyses.

### Characteristics of the PDAP episodes

The baseline characteristics of the enrolled patients are presented in Tables 1 and 2. The average age was 50.5 years, and 259 (53.4%) of the patients were male. The median PD vintage was 25.7 months. There were 84 patients (17.3%) with DM and 114 patients (23.5%) with a history of CVD, and the average CCI score was 4.3. Regarding pathogenic organisms, 167 (34.4%) were gram-positive bacteria, 48 (9.9%) were gram-negative bacteria, 23 (4.7%) were fungi, 3 (0.6%) were mixed infections, and 244 (50.3%) were culture-negative. Most patients only had one episode of peritonitis, and the technique failure group had higher proportion of patients who experienced 2 or more episodes of peritonitis.

**Table 1:** Baseline demographic characteristics

vaiables	Total (*n* = 485)	Technique survival group (*n* = 355)	Technique failure group (*n* = 130)	*P* value*
Sex, male, *n* (%)	259 (53.4)	187 (52.7)	72(55.4)	0.596
Age, year	50.5 ± 14.0	50.3 ± 14.0	51.2 ± 14.0	0.529
Residence, city, *n* (%)	258 (53.2)	192 (54.1)	66 (50.1)	0.517
PD vintage, months	25.7 (12.6, 54.1)	25.2 (11.5, 48.8)	36.8 (15.7, 69.9)	0.002
DM, *n* (%)	84 (17.3)	61 (17.2)	23 (17.7)	0.896
CVD, *n* (%)	114 (23.5)	73 (20.6)	41 (31.5)	0.012
CCI score, points	4.3 ± 1.9	4.2 ± 1.9	4.6 ± 2.0	0.050
SBP, mmHg	135.5 ± 25.2	135.3 ± 25.0	136.0 ± 26.0	0.791
DBP, mmHg	84.2 ± 16.4	84.2 ± 16.3	84.2 ± 117.0	0.996
Hospital stay, days	15.0 (11.0, 21.0)	14.0 (10.0, 18.0)	21.0 (15.0, 29.3)	< 0.001
PDAP episodes				0.163
1 episode, *n* (%)	426 (87.8%)	317 (89.3%)	109 (83.8%)	
2 episodes, *n* (%)	51 (10.5%)	34 (9.6%)	17 (13.1%)	
≥ 3 episodes, *n* (%)	8 (1.6%)	4 (1.1%)	4 (3.1%)	

Notes: Values are expressed as the mean ± SD, *n* (%), or median (Q1–Q3)

*, Comparison between technique failure group and technique survival group

### Comparison between technique failure group and technique survival group

There were 355 episodes with technique survival (73.2%) and 130 episodes with technique failure (26.8%). To explore the potential risk factors, we conducted a univariate analysis (Tables 1 and 2). The PD vintage and hospital stay in the technique failure group were significantly longer than those in the technique survival group (*P* = 0.002 and *P* < 0.001, respectively), and the incidence of CVDs was significantly higher in the technique failure group (*P* = 0.012).

As shown in Tables 2 and 3, ALB, BUN, and Na levels were significantly higher in the technique survival group than in the technique failure group (*P* < 0.05). The technique failure group had higher levels of WBC, PLT, neutrophils, CHOL, NT-proBNP, FIB, APTT, PT, and ferritin (*P* < 0.05). The peritoneal dialysate white cell counts in the technique failure group on days 3 and 5 were significantly higher than those in the technique survival group (*P* < 0.05). The proportions of fungal and multiple microbial infections were significantly higher in the technique failure group (*P* < 0.001 and *P* = 0.004, respectively). Compared to the technique survival group, the technique failure group displayed higher NT-proBNP levels for each category of infecting pathogen, including the culture-negative, gram-positive bacteria, and gram-negative bacteria groups, with statistically significant differences. However, within the technique survival and technique failure groups, we found no differences in NT-proBNP levels among the causative organisms.

**Table 2:** Baseline characteristics of laboratory variables

Variables	Total (*n* = 485)	Technique survival group (*n* = 355)	Technique failure group (*n* = 130)	*p* value*
HB, g/L	91.9 ± 20.7	93.0 ± 20.92	88.9 ± 19.7	0.055
PLT, 10^9^/L	192.0 (144.0, 262.0)	188.0 (138.0, 255.0)	216.5 (163.0, 302.8)	0.001
WBC, 10^9^/L	7.1 (5.7, 9.5)	6.7 (5.3, 9.0)	8.0 (6.2, 11.6)	0.001
Neutrophil, 10^9^/L	5.5 (4.0, 7.7)	5.0 (3.8, 7.2)	6.27 (4.6, 9.3)	< 0.001
DB, µmol/L	1.9 (1.3, 2.6)	1.8 (1.3, 2.5)	2 (1.2, 3.1)	0.128
IB, µmol/L	2.9 (2, 4.25)	3.0 (2.2, 4.3)	2.4 (1.6, 3.6)	< 0.001
ALB, g/L	29.2 ± 6.4	30.1 ± 6.4	26.8 ± 6.0	< 0.001
GLB, g/L	27.90 (24.00, 32.00)	27.10 (27.90, 32.30)	29.1 (24.78, 34.2)	0.009
GLU, mmol/L	5.5 (4.6, 7.0)	5.4 (4.6, 7.2)	5.8 (4.9, 7.5)	0.267
BUN, mmol/L	17.2 (12.9, 21.4)	17.2 (13.1, 21.6)	16.4 (12.0, 20.8)	0.049
SCr, µmol/L	880.6 ± 305.1	883.0 ± 301.4	874.0 ± 316.1	0.774
eGFR, ml/(min*1.73m^2^)	5.1 (4.1, 6.6)	5.1 (4.2, 6.6)	4.9 (3.9, 6.5)	0.280
UA, µmol/L	353.3 ± 87.4	353.9 ± 79.5	351.6 ± 106.4	0.795
TG, mmol/L	1.3 (0.9, 2.0)	1.3 (0.9, 2.0)	1.4(1.0, 2.1)	0.091
CHOL, mmol/L	4.0 ± 1.1	4.1 ± 1.2	3.7 ± 1.0	< 0.001
LDL-C, mmol/L	2.24 ± 0.95	2.34 ± 0.97	1.98 ± 0.88	< 0.001
Na, mmol/L	138.1 ± 4.2	138.6 ± 4.1	136.7 ± 4.2	< 0.001
K, mmol/L	3.78 ± 0.7	3.8 ± 0.7	3.7 ± 0.7	0.121
Ca, mmol/L	2.16 (2.01, 2.28)	2.15 (2.01, 2.29)	2.16 (1.99, 2.28)	0.497
P, mmol/L	1.38 (1.09,1.74)	1.36 (1.09,1.72)	1.40 (1.76, 1.09)	0.868
Ferritin, ng/mL	267.6 (176.7, 486.8)	242.9 (156.3, 471.0)	328.6 (190.6, 612.6)	0.003
iPTH, pmol/L	21.3 (9.3, 37.1)	20.4(8.0, 34.4)	22.4 (11.5, 41.4)	0.100
PT, s	12.20 (11.50, 13.10)	12.00(11.50, 13.00)	12.40 (11.88, 13.40)	0.009
APTT, s	30.00 (26.80, 33.60)	29.10(26.30, 32.88)	31.30 (28.55, 36.88)	< 0.001
FIB, g/L	5.08 (4.46, 6.19)	4.83 (4.33, 6.04)	5.56 (4.95, 6.59)	< 0.001
NT-proBNP, pg/mL	9000.0 (4339.5, 17219.0)	6872.0 (3495.0, 12379.0)	17167.5 (10378.8, 23876.0)	< 0.001
Peritoneal dialysate white cell counts				
Day 1, 10^6^/L	1240.0 (292.5, 3982.5)	990.0 (285.0, 3600.0)	1400.0 (290.0, 3790.0)	0.775
Day 3, 10^6^/L	280.0 (70.0, 1034.0)	140.0 (40, 661.8)	799.5 (267.8, 2051.3)	< 0.001
Day 5, 10^6^/L	70.0 (12.0, 360.0)	30.0 (10.0, 225.0)	340.0 (80.0, 715.0)	< 0.001
Causative organisms				
Culture negative	244 (50.3%)	183 (51.5%)	61 (46.9%)	0.367
Gram-positive bacteria	167 (34.4%)	128 (36.1%)	39 (30.0%)	0.214
Gram-negative bacteria	48 (9.9%)	34 (9.6%)	14 (10.8%)	0.697
Fungal	23 (4.7%)	10 (2.8%)	13 (10.0%)	0.001
Polymicrobial	3 (0.6%)	0 (0.0%)	3 (2.3%)	0.004

Notes: Values are expressed as the mean ± SD, n (%), or median (Q1–Q3). *, Comparison between technique failure group and technique survival group

**Table 3:** Comparison of NT-proBNP levels based on causative organism

	NT-proBNP level			
	Total (*n* = 485)	Technique survival group (*n* = 355)	Technique failure group (*n* = 130)	*P* value
Causative organisms				
Culture negative (*n* = 244)	8250 (4120, 15,910)	6960 (3460, 10,550)	17,570 (8630, 25,440)	< 0.001
Gram-positive bacteria (*n* = 167)	8740 (4300, 18,250)	6760 (3590, 12,770)	18,470 (13,790, 31,740)	< 0.001
Gram-negative bacteria (*n* = 48)	1030 (4690, 15,910)	9570 (3830, 11,390)	13,400 (6820, 20,420)	0.045
Fungal (*n* = 23)	13,130 (8450, 29,790)	9770 (4670, 23,070)	14,420 (11,980, 31,690)	0.192
Polymicrobial (*n* = 3)	17,370 (3970, 18,780)	—	17,370 (3970, 18,780)	—
*P* value	0.169	0.544	0.438	

Multivariate analyses indicated that hospital stay (OR, 1.056; 95% confidence interval [CI], 1.031-1.081; *P* < 0.001), Na (OR, 0.927; 95% CI, 0.865-0.994; *p* = 0.033), NT-proBNP (OR, 1.062; 95% CI, 1.035-1.090, *P* < 0.001), peritoneal dialysate white cell counts on day 3 (OR, 1.222; 95% CI, 1.030-1.449, *P* = 0.022) and on day 5 (OR, 2.367; 95% CI, 1.396-4.013, *P* = 0.001) were independently associated with treatment failure (Table 4).

**Table 4:** Multivariate analysis of risk factors of technique failure

Variable	OR (95% CI)	*P* value
Na, mmol/L	0.927 (0.865, 0.994)	0.033
Hospital stay, days	1.056 (1.031, 1.081)	< 0.001
NT-proBNP, per 10^3^pg/mL increase	1.062 (1.035, 1.090)	< 0.001
Peritoneal dialysate white cell counts on day3, per 10^3^/ul increase	1.222 (1.030, 1.449)	0.022
Peritoneal dialysate white cell counts on day5, per 10^3^/ul increase	2.367 (1.396, 4.013)	0.001

### ROC curves for NT-proBNP index and technique failure risk in PDAP

**Fig. 2:**
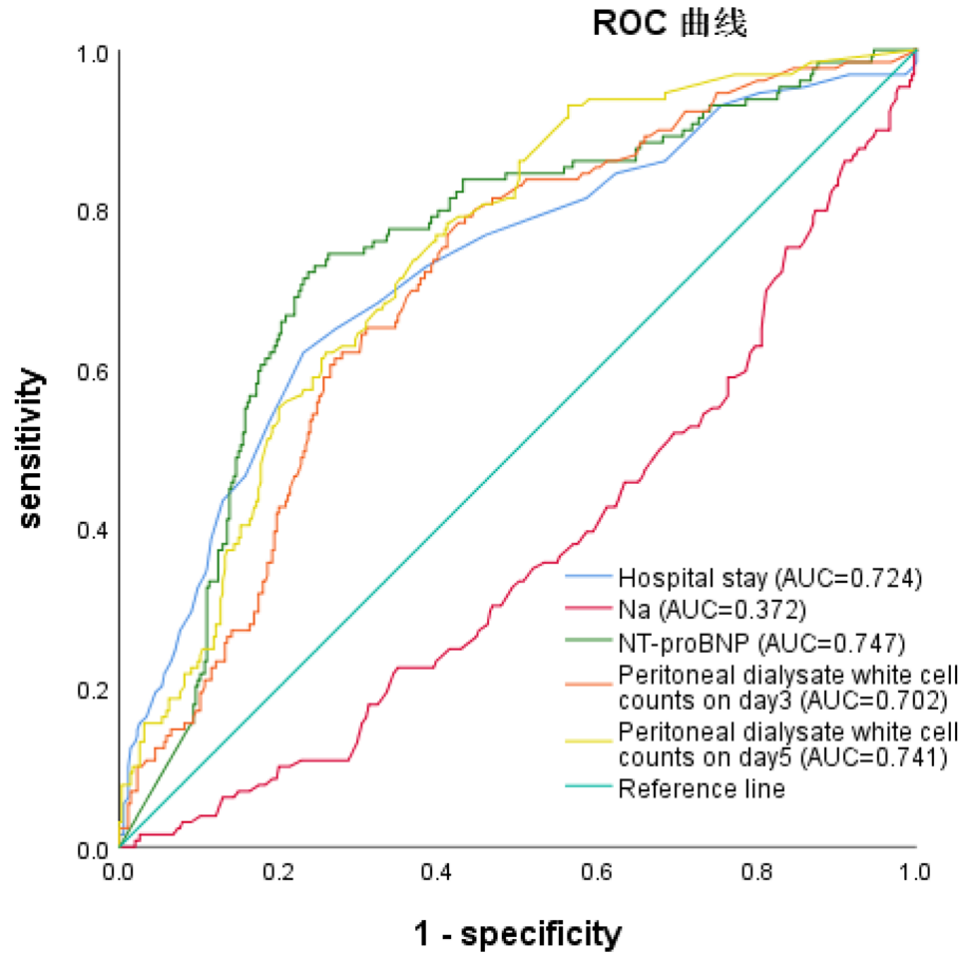
Receiver operating characteristic curves for the risk factors of treatment failure in peritoneal dialysis related peritonitis

According to the ROC analysis, the NT-proBNP level was superior to hospital stay, Na, and peritoneal dialysate white cell counts on days 3 and 5 for indicating technique failure. The optimal cut-off value for NT-proBNP was 11,392 ng/L, with a Youden index of 0.486, sensitivity of 73.1%, and specificity of 75.5% (area under the curve [AUC], 0.747; 95% CI, 0.697, 0.797) (Fig. 2; Table 5).

**Table 5:** ROC curve analysis. Notes: AUC area under the curve; CI confidence interval; ROC receiver operating characteristic

Variable	AUC	95%CI	*P* value	Sensitivity	Specificity	F1-score	cutoff
NT-proBNP	0.747	0.697, 0.797	< 0.001	73.1%	75.5%	0.486	11,392
Na	0.372	0.316, 0.427	< 0.001	55.0%	24.8%	-0.202	135.95
Hospital stay	0.724	0.670, 0.777	< 0.001	62.3%	76.9%	0.392	18.5
Peritoneal dialysate white cell counts on day3	0.702	0.652, 0.752	< 0.001	77.7%	57.5%	0.352	255
Peritoneal dialysate white cell counts on day5	0.741	0.694, 0.788	< 0.001	78.5%	58.9%	0.374	63

Notes: AUC area under the curve; CI confidence interval; ROC receiver operating characteristic

### NT-proBNP and technique failure risk in PDAP

Patients with PDAP episodes were allocated to one of two groups according to the median plasma BNP level: a low NT-proBNP group (plasma NT-proBNP < 9000 ng/L) and a high NT-proBNP group (plasma NT-proBNP ≥ 9000ng/L). The hazard ratios of NT-proBNP associated with PDAP from the adjusted Cox proportional hazard models are listed in Table 6. Cox regression analysis showed that the high NT-proBNP group had an increased risk of technique failure, with an HR of 3.020 (95% CI 1.771, 5.150, *P* < 0.001 compared with the low NT-proBNP group. Regardless of the adjustment model used, NT-proBNP (per-unit increase) and NT-proBNP ≥ 9000 (vs. NT-proBNP < 9000) independently increased the risk of technique failure in PDAP (Table 6).

**Table 6:** Adjusted and unadjusted Hazard Ratio for Cox proportional hazard models

NT-proBNP			NT-proBNP ≥ 9000ng/L (vs. NT-proBNP <9000ng/L)	
	HR (95% CI)	*p* value	HR (95% CI)	*p* value
Unadjusted	1.027 (1.011, 1.043)	0.001	2.658 (1.660, 4.255)	< 0.001
Model 1	1.027 (1.010, 1.043)	0.001	2.701 (1.684, 4.331)	< 0.001
Model 2	1.025 (1.008, 1.043)	0.004	2.765 (1.680, 4.549)	< 0.001
Model 3	1.030 (1.009, 1.051)	0.004	3.020 (1.771, 5.150)	< 0.001

Notes: model1: age, sex

model2: model1 plus history of diabetes, history of CVD, the CCI score, PD duration, SBP, DBP

model3: model2 plus HB, WBC, neutrophil, PLT, IB, ALB, GLB, CHOL, LDL-C, Na, iPTH, APTT, PT, FIB, ferritin, Peritoneal dialysate white cell counts on day 3 and 5

## Discussion

An expanding body of research has focused on identifying the risk factors associated with adverse outcomes of PDAP. The early recognition of these factors can inform treatment strategies and improve patient prognosis. This study used a retrospective cohort design to investigate the relationship between NT-proBNP levels and technical failure in patients with PDAP. The findings revealed that higher serum NT-proBNP levels at the onset of PDAP were independently associated with technical failure within 30 days of peritonitis. After adjusting for confounding variables such as eGFR, sex, age, diabetes mellitus and cardiovascular disease, an elevated serum NT-proBNP level was still found to independently indicate serious outcomes, establishing the admission NT-proBNP level as an independent risk factor of technique failure in patients with PDAP.

NT-proBNP is an inactive amino-terminal fragment derived from the cleavage of pro-BNP and consists of 108 amino acids. The release of BNP counteracts the effects of the renin-angiotensin-aldosterone system [10–12]. The measurement of both BNP and NT-proBNP in plasma and serum serves as a valuable diagnostic and prognostic tool for management of congestive heart failure and other cardiovascular diseases [13, 14]. In general, BNP and NT-proBNP levels demonstrate a reasonable correlation, with NT-proBNP levels reflecting BNP levels. However, NT-proBNP has the advantages of a longer plasma half-life and lower biological variation than BNP [10, 15]. The half-life of BNP is approximately 20 min, whereas that of NT-proBNP extends to approximately 1–2 h, resulting in elevated circulating levels—the circulating NT-proBNP level is approximately six-fold higher than that of BNP—and slower fluctuations compared to BNP, despite their equal secretion ratios [10, 16]. As such, NT-proBNP might provide a superior index of dysfunction than BNP alone [17].

Wang et al. demonstrated the significant prognostic value of NT-proBNP in terms of cardiovascular congestion, mortality, and cardiovascular death in patients with chronic PD [18]. Furthermore, they suggested that the extent of NT-proBNP elevation in patients with chronic PD may, in part, reflect extracellular volume expansion rather than merely serve as a biomarker for left ventricular hypertrophy and systolic dysfunction [19]. Over half of patients with PD experience fluid overload [20, 21]. Previous studies have also established positive correlations among changes in NT-proBNP levels, extracellular fluid volume, total body water, and the ratio of extracellular fluid volume to total body water in dialysis patients [22]. Additional studies have demonstrated that increased NT-proBNP levels are associated with hypervolemia in patients on dialysis [11, 12].

Inflammation may contribute to the development of hypervolemia [12, 19, 23]. Consequently, patients with peritonitis are at higher risk of developing hypervolemia. Inflammation can increase peritoneal permeability, characterized by higher peritoneal transport, resulting in a gradual elevation in NT-proBNP levels, potentially attributable to heightened glucose reflux from the abdominal cavity into the bloodstream, augmented fluid reabsorption rates, diminished osmotic gradients, and reduced ultrafiltration volumes [24–27]. The urine volume may also decrease sharply during this course in the paitents with residual renal function. Consequently, this population is more susceptible to the perils of volume overload, such as heart failure, subsequent hemodialysis, and even mortality. Therefore, serial assessments of NT-proBNP levels may prove beneficial for evaluating the volume status in patients with PDAP, thereby assisting in clinical decision making to enhance technique outcomes.

In our study, we identified several factors independently associated with technique failure, including hospital stay, sodium levels, NT-proBNP levels, and peritoneal dialysate white cell counts on days 3 and 5. Among these factors, NT-proBNP showed the best sensitivity and accuracy as indicated by the largest area under the curve. We constructed three risk models, each with various confounding factors. The NT-proBNP level remained an independent indicator of technical failure in PDAP in all models. This finding highlights the importance of monitoring and managing fluid volume, including the NT-proBNP level and volumes of ultrafiltration and urine, in patients with PDAP. It is crucial not only to recognize and address volume overload but also to appropriately adjust peritoneal dialysis prescriptions to prevent persistent volume overload, which can lead to cardiac complications and increased mortality.

Our study had several limitations that should be acknowledged. First, it was a retrospective study conducted at a single center, which may have introduced bias into our findings. Therefore, it is necessary to conduct further large-scale cohort studies to better understand and validate these results. In addition, it is important to conduct further investigations to examine the pathogenesis of NT-proBNP levels in patients with PDAP. Second, certain variables such as IL-6, hs-CRP, and PCT were excluded because of high rates of missing data. Exploring the combination of these variables with NT-proBNP levels may provide more accurate prognostic values and should be considered in further studies. Third, although cardiovascular disease was included in risk model 2, we did not consider the presence of cardiac structural abnormalities, such as mitral insufficiency, which may influence NT-proBNP levels independently of fluid status. Furthermore, our data collection fell short in encompassing additional metrics for evaluating volumetric equilibrium, such as edema, weight fluctuations, detailed ultrafiltration, and extravascular lung water. This may potentially lead to inaccuracies in volume evaluation and volume control. Finally, the proportion of negative culture results was high. Notably, many of our patients live far from our hospital, and they have been prescribed antibiotics by local doctors before admission. The training of regional primary care physicians should therefore be strengthened, while simultaneously contemplating next-generation sequencing as a viable option for etiological diagnosis.

## Conclusions

Our study identified a significant association between admission serum NT-proBNP levels and the risk of technique failure in patients with PDAP. Furthermore, the NT-proBNP level emerged as an independent risk factor for technique failure in these patients. These findings emphasize the importance of measuring NT-proBNP levels during PDAP and adjusting the volume status accordingly could potentially improve the prognosis of patients with PDAP.

## Data Availability

No datasets were generated or analysed during the current study.

## Abbreviations

PD: Peritoneal dialysis
ESRD: End-stage renal disease
PDAP: Peritoneal dialysis-associated peritonitis
CRP: C-reactive protein
NT-proBNP: N-terminal fragment of B-type natriuretic peptide
ISPD: International Society for Peritoneal Dialysis
SBP: Systolic blood pressure
DBP: Diastolic blood pressure
DM: Diabetes mellitus
CVDs: Cardiovascular diseases
CCI: Charlson Comorbidity Index
HB: Hemoglobin
WBC: White blood cell, neutrophil
PLT: Platelet
DB: Direct bilirubin
IB: Indirect bilirubin
ALB: Serum albumin
GLB: Serum globulin
GLU: blood glucose
BUN: Blood urea nitrogen
Scr: Serum creatinine
eGFR: Estimated glomerular filtration rate
UA: Uric acid
TG: Triglycerides
CHOL: Total cholesterol
LDL-C: Low-density lipoprotein cholesterol
Na: Serum sodium
K: Serum potassium
CA: Serum calcium
P: Serum phosphorus
iPTH: intact parathyroid hormone
APTT: Activated partial thromboplastin time
PT: Prothrombin time
FIB: Fibrinogen
PMN: Polymorphonuclear leukocytes
ROC: Receiver operating characteristic
CI: Confidence interval
AUC: Area under the curve
